# Disease Load at Conception Predicts Survival in Later Epidemics in a Historical French-Canadian Cohort, Suggesting Functional Trans-Generational Effects in Humans

**DOI:** 10.1371/journal.pone.0093868

**Published:** 2014-04-16

**Authors:** Kai Willführ, Mikko Myrskylä

**Affiliations:** 1 Max Planck Institute for Demographic Research, Rostock, Germany; 2 The London School of Economics and Political Science, London, United Kingdom; National Institute of Environmental Health Sciences, United States of America

## Abstract

**Objective:**

Functional trans-generational and parental effects are potentially important determinants of health in several mammals. For humans, the existing evidence is weak. We investigate whether disease exposure triggers functional trans-generational response effects among humans by analyzing siblings who were conceived under different disease loads, and comparing their mortality in later epidemics. Under functional trans-generational response mechanisms, we expect that those who were conceived under high pathogenic stress load will have relatively low mortality during a later epidemic.

**Methods:**

We use data from the *Registre de la Population du Québec Ancien*, which covers the historical population living in St. Lawrence Valley, Québec, Canada. Children born in 1705–1724 were grouped according to their exposure during conception to the measles 1714–15 epidemic. The 1714–15 epidemic was followed by two mortality crises in 1729–1734. The cause of the first crises in 1729 is not exactly known. The second crisis in 1732 was caused by a smallpox epidemic. Using proportional hazard Cox regression models with multivariate adjustment and with fixed-effects approach that compare siblings, we analyze whether mortality in 1729–1734 is affected by exposure to the 1714–15 epidemic.

**Results:**

Children who were conceived during the peak of the measles epidemic of 1714–15 exhibited significantly lower mortality during the 1729–1734 crisis than those who were born before the 1714–15 epidemic (mortality hazard ratio 0.106, p<.05 in multivariate adjusted models; 0.142 p<.1 in sibling comparison models).

**Conclusions:**

The results are consistent with a trans-generational mechanism that functionally responds to pathogen stress and suggest that early disease exposure may be protective later in life. Alternative explanations for the mortality patterns are discussed and shown to be problematic.

## Introduction

Trans-generational and parental effects are widespread in the animal kingdom. For instance, it has shown that mammals exhibit trans-generational effects when fed special diets [1], under exposure to certain chemicals [2], [3], or when they have undergone traumatic experiences [4], [5]. Thus parental environmental conditions appear to influence their offspring’s phenotype via different pathways including epigenetic inheritance. This finding could have implications for public health.

Some trans-generational effects can be interpreted as functional mechanisms, because they reflect an adaptation or a co-adaptation, and they respond to environmental cues in a functional manner. For example, water fleas (*Daphnia spec.*) are able to initiate trans-generational, predator-specific defense mechanisms [6], [7]. As a heuristic approach, it is useful to separate those functional trans-generational effects from non-functional ones which do not serve any biological purpose [8]. While there is considerable evidence for functional trans-generational effects among non-human species, the practical importance of these findings for humans is inconclusive. There are trans-generational effects in humans [9]–[11], but the functional interpretation of these effects is unclear. A study examining the historical population of the St. Lawrence Valley in Quebec, Canada compared the mortality of children that were conceived under different pathogenic environments based on region of residence and found no evidence of functional effects or epigenetic inheritance [12]. However, most biological response mechanisms are cue- and context -specific. Therefore, negative results in one context do not imply that functional trans-generational effects do not occur in general.

When applied to humans, the concept of functional trans-generational effects has been criticized. The main argument of these critics is that cost-benefit calculations would often be negative. This is because decades generally elapse between the conception of a child and the start of his or her own reproduction, which means that parental predictions about a child’s future environment are often incorrect. This could result in a phenotype-environment mismatch; compare the so-called *Predictive Adaptive Response Theory* [13]–[15]. However, functional trans-generational effects do not have to affect the offspring’s adult phenotype; they could also affect the child’s juvenile development, by, for example, altering the resource allocation between growth and maintenance.

In this study, we test the hypothesis that increased pathogen or epidemic events are linked to parental effects that functionally modify offspring’s phenotype. The concept of functional trans-generational response mechanisms predicts that the altered imprinting of the offspring’s epigenome will result in a phenotype which is less vulnerable under high levels pathogenic stress, but which may exhibit lower levels of fitness if the subsequent disease load is low. In some senses, it is expected that these offspring will be “pre-adapted” or “pre-adjusted” to environments with high levels of pathogenic stress. We used data from the *Registre de la population du Québec ancien* on the historical population of the St. Lawrence Valley (Québec, Canada) in order to investigate whether the children’s exposure to different levels of pathogenic stress during conception affected their mortality in later epidemics, as well as in periods of lower disease loads.

**Figure 1:**
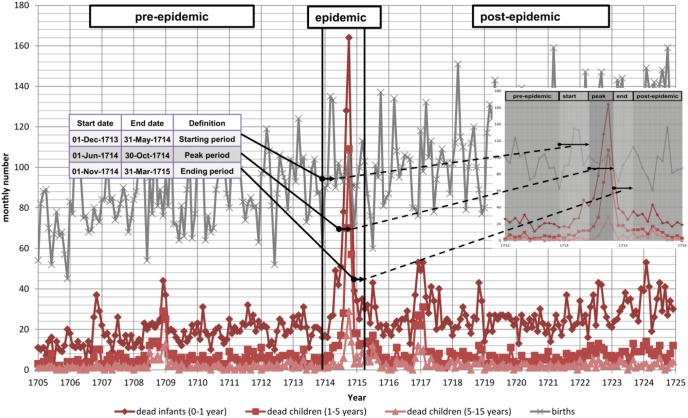
Monthly number of births, dead infants (0–1 year), small children (1–5 years), and children (5–15 years) from 1705 until 1724, and classification of the measles epidemic 1714–15 into a pre-epidemic, starting, peak, ending, and post-epidemic period.

**Figure 2:**
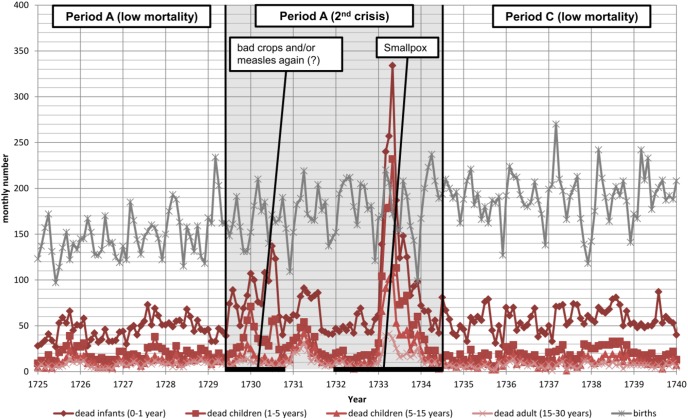
Graph showing the monthly number of births, dead infants (0–1 year), small children (1–5 years), children (5–15 years), and young adults (15–30 years) from 1725 until 1740. The period between Jun. 1, 1729 and Jun. 30, 1735 is characterized by two periods of increased mortality in quick succession and is considered as a single crisis period.

The population of the St. Lawrence Valley was periodically hit by epidemics of measles, smallpox, typhus, and other diseases. For the purposes of this study, we needed to identify a natural experiment situation in which a distinct epidemic was followed by a period of rather low and stable mortality, and which was then followed by another epidemic. Such a setting can be observed between 1705 and 1740. From 1705 until the middle of 1714, mortality was more or less stable. In 1714–15, a severe measles epidemic spread. Infants and toddlers were especially affected [16], [17] (see Figure 1). The measles epidemic was then followed by 14 years of rather low and stable mortality. From 1729 to 1734, two mortality crises struck in quick succession, which in this study were considered as a single contiguous crisis period (see Figure 2). The cause of the first, smaller crisis that occurred in 1729 is not exactly known, but it may have been caused by a measles epidemic and/or by bad crops [17]. The second crisis in 1732 was caused by a smallpox epidemic [17]. Mortality among infants, children, and young adults aged 15 to 30 rose sharply during the 1729–34 crisis. This cohort included individuals who were conceived around the measles epidemic of 1714–15. The period between 1735 and 1740 was characterized by a low and stable mortality rate. We also analyzed the two crises separately (data not shown). The results do not substantially differ across these periods (Jun. 01, 1729 to Dec. 31, 1731 and Jan. 01, 1732 to Jun. 30, 1734). However because of small sample size the effects were statistically not significant when these periods were treated separately. Therefore, the period of Jun. 01, 1729 to Jun. 30, 1734 is considered as one continuous crisis period.

## Study Population and Period

### The Population of the St. Lawrence River in Québec, 1705–1740

We analyzed the historical population of the St. Lawrence River in Québec, Canada (1621–1799). The data came from the *Registre de la population du Québec ancien* (RPQA), which was created by the *Programme de recherche en démographique historique* (PRDH) at the University of Montreal.

Another reason why we chose the period 1705–1740 for our study was that during the preceding time periods, the population was naturally fertile and was relatively undisturbed by other populations. At the beginning, the colony was very small and suffered from a shortage of women [18]. Because of this shortage, only a few children were born in the colony; the majority of the inhabitants were born in France. In 1666, when the first census was taken, around 3,200 people were living in the colony [19]. After an immigration wave in 1674, the colony experienced an exponential growth in population which was mainly caused by births within the colony. Around 1740, there were approximately 55,000 inhabitants. Likewise, the time period after 1750 appeared unsuitable because the population faced a worsening of their living conditions, which resulted in an increase in infant and child mortality. The main reason for this worsening of conditions was the French and Indian War (1754–1763) and its consequences for the population of New France, which became part of the British Empire after the war.

We included children who were born in 1705–1724 (N = 28,035). We selected only children with a father and a mother who were not married before the marriage in which the child was born (5,376 cases deleted). In recomposed families, the levels of parental investment often vary because remarriage alters kinship relations (step-parenthood, et cetera [20]). Especially during an epidemic, a child’s mortality risk may be biased if childcare is provided by a non-biologically-related parent. We used methods that compare siblings with different epidemic exposures. Therefore we included only sibling sets in which at least on child was born during the epidemic and one another prior or after the epidemic (N = 10,614 cases deleted). After exclusion of cases with missing data on the parent’s birth and death dates or residential status, our remaining sample size was 7,947 children from 575 families.

We use information on the children’s sex, birth rank, and birth and death dates which are all directly available in the data. We estimate the conception date assumption that the pregnancies lasted 9 months. We also use information on the parents’ ages at death, their residential status, and ages at the child’s birth.

## Methods

**Figure 3:**
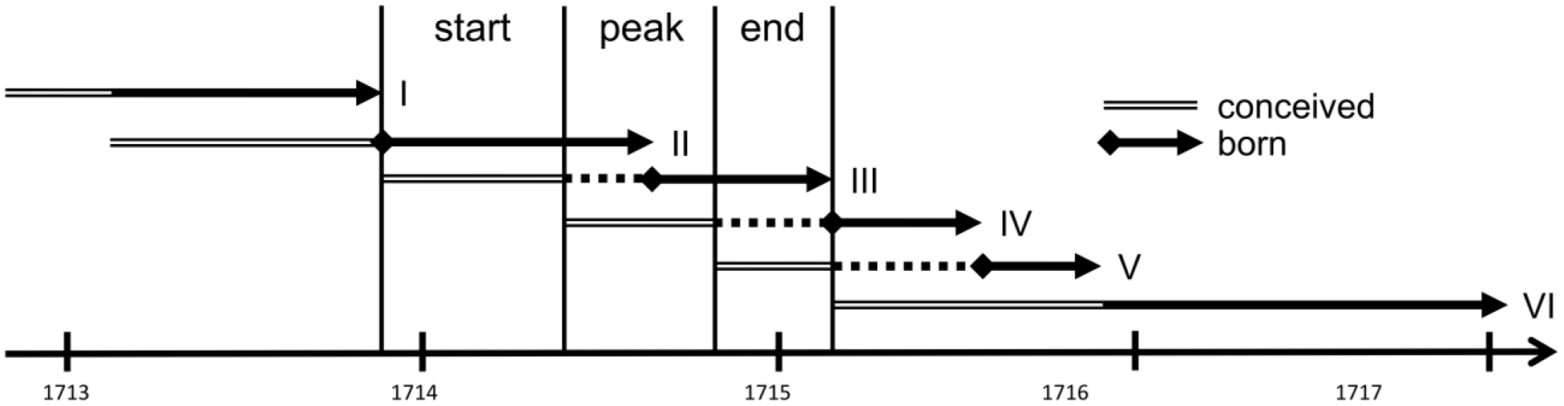
Time line illustrates the chronology of the six exposure statuses during conception.

We divided the 1714–15 measles epidemic into three exposure periods: starting (Dec. 01, 1713–May 31, 1714), peak (Jun. 01, 1714–Oct 30, 1714), and ending period (Nov. 01, 1714–Feb. 28, 1715) (see Figure 1). This classification, in particular the peak period, corresponds to the death rates reported elsewhere [16], [17]. We split the data into six categories based on these three exposure periods: I, conceived and born prior to the epidemic (N = 3,467); II, conceived before but born during the epidemic (N = 362); III, conceived during the beginning and born in the peak or end period of the epidemic (N = 241); IV, conceived during the peak and born after the epidemic (N = 200); V, conceived during the end period and born after the epidemic (N = 156); and VI, conceived and born after the epidemic (N = 3,521) (see time line in Figure 3).

We used Cox proportional hazards regression models [21] to estimate the mortality of children during three time periods: Jan. 01, 1725 to May 31, 1729 (A, the period before the second crisis); Jun. 01, 1729 to Jun. 31, 1734 (B, the second crisis period); and Jul. 01, 1734 to Dec. 31, 1740 (C the period after the second crisis) (see Figure 2). For each time period, we estimated two models. The first, Model I, was a standard multivariate model that controlled for age and age squared, sex, birth rank, mother’s and father’s ages at death, their ages at the child’s birth, and their residential status. These factors have all been linked to mortality, as parental life span is inheritable [22], child survival is affected by sex and birth order [23], child mortality increased in the colony of the St. Lawrence Valley over time [19], maternal and paternal ages are associated with survival [24], [25]. Moreover, Mazan [17] has shown that immigrants had higher mortality in the measles epidemic of 1714–15, which is why we controlled for mother’s and father’s residential status and mortality varies by residential status.

Model II was a fixed-effects model which was based on sibling comparison, and which controlled for age, age squared, sex, birth rank and mother’s age at the child’s birth. The fixed-effect Cox model allows the baseline hazard to be family-specific. As a result, through sibling comparison all observed and unobserved environmental and biological factors that were shared by the siblings are controlled for in the model [26]. For example, characteristics such as parental socioeconomic status, cognitive ability or parental personality are, to the extent they do not vary between siblings, controlled. Time-varying and non-shared factors (such as birth order or parental age) are not automatically captured by the model. Consequently, we included controls for the observed non-shared factors of age, age squared, sex, birth rank, and mother’s age at the child’s birth.

Models I and II may deliver different results because of they control for a different set of background variables. Model I, which compares persons across families, controls only for those variables observed in the data. Children from different families may differ not just according to observed characteristics but also according to large number of unobserved variables. Model II controls for these unobserved social and biological characteristics that are shared by the siblings. Therefore, Model II is less prone to unobserved variable bias. When results across Model I and Model II differ, emphasis should be based on Model II.

In all models, category I (the child was conceived and born before the 1714–15 measles epidemic) was set as reference group and the disease exposure effect was estimated by including indicators for the other exposure groups. Further, category I covers a longer time period (Jan. 01, 1705–Dec. 31, 1713) and therefore includes more individuals than the exposure categories II, III, IV and V which only cover a couple of months. The length of category I make it less sensitive to short-term fluctuations which might occur for unknown reasons.

This approach of comparing the reference group of non-exposed individuals (category I) to those with some measles exposure is not dependent on an assumption that every individual of the exposure categories (category II, III, IV and V) exposed. If a fraction of a category was exposed and exhibit significantly different survival, standard statistical reasoning implies our models underestimate the true effect. However, the severity of the measles epidemic of 1714–15 led to every parish of the St. Lawrence valley being affected [17]. Given this, it is likely that nearly every person living during that time period, resistant/immune or vulnerable, had contact with infected individuals.

## Results

### Descriptive Results

Figure 1 shows the absolute monthly numbers of births, dead infants and children over the period of 1705–24. The graphs indicates that mortality peaked during what we define the peak 1714–15 epidemic period. The vertical lines illustrating the defined borders of five different periods of the measles epidemic. Based on these, we spilt the children born between 1705 and 1724 into six difference exposure categories (see also Figure 3). Figure 2 shows the crude mortality rates over three later periods (1725–29; 1729–34; 1734–40) for which we estimate mortality by exposure status. The graphs shows that mortality peaked again in the 1729–34 crisis years, but was relatively low before and after the years 1729–34. Note that we used absolute monthly numbers of deaths to identify periods of increased mortality. As discussed above the population experienced an exponential population growth during the study period. Therefore, a simple comparison of crises (e.g. between 1714–15 and 1729–34) regarding their strength by crude death counts is not possible if they did not occur in quick succession.

Table 1 shows the sample size by exposure category and the numbers of deaths by exposure category and the three later periods: before the 1729–34 crisis years (A); the 1729–34 crisis years (B); and after the 1729–34 crisis years (C). In the period A 4.17% of those who were alive in the beginning of the period died; this proportion was lowest (1.34%) in the group that was conceived before and born during the 1714–15 epidemic (category II) and highest (4.55%) for those who were conceived and born after the epidemic (category VI). During the second crisis period 6.08% of those who survived to the baseline died. This proportion varied from 7.22% among those who were conceived before and born before or during the 1714–15 epidemic (categories I, II) to 0.71% among those who were conceived during the peak of the epidemic (category III). These results tentatively suggest that being conceived during the epidemic might have been protective in the later crisis years. In the period 1735–40 further 4.69% of the survivors died; this proportion varied only little by exposure status.

**Table 1:** Cases remaining after data selection.

Mortality during period		A (Jan. 01, 1725–May 31, 1729)			B (2^nd^ crisis) (Jun. 01, 1729–Jun. 30, 1735)			C (Jul. 01, 1735–Dec. 31, 1740)		
Exposure status	N births	N alive at Jan. 01, 1725	N dead at May 31, 1729	% died	N alive at Jun. 01. 1729	N dead at Jun. 30, 1735	% died	N alive at Jan. 01, 1735	N dead at Dec. 31, 1740	% died
**I** conceived & born before the epidemic (REF) [born Jan. 01, 1705–Dec. 31, 1713]	3,467	2,531	107	4.23	2,424	175	7.22	2,249	120	5.34
**II** conceived before, born in the epidemic [born Jan. 01, 1714–Aug. 31, 1714]	362	224	3	1.34	221	16	7.24	205	9	4.39
**III** conceived in the start, born in the epidemic [born Sep. 01, 1714–Feb. 28, 1715]	241	175	3	1.71	172	10	5.81	162	7	4.32
**IV** conceived in the peak, born after the epidemic [born Mar. 01, 1715–Jul. 31, 1715]	200	146	6	4.11	140	1	0.71	139	7	5.04
**V** conceived in the end, born after the epidemic [born Aug. 01, 1715–Nov. 30, 1715]	156	123	4	3.25	119	4	3.36	115	8	6.96
**VI** conceived & born after the epidemic [born Dec. 01, 1715–Dec. 31, 1724]	3,521	2,723	124	4.55	2,599	139	5.35	2,460	99	4.02
**Sum**	7,947	5,922	247	4.17	5,675	345	6.08	5,330	250	4.69

Given are the total numbers of cases of the six exposure categories that remain after data selection (N births), as well as the number of individuals who survived until Jan. 01, 1725 (A), Jun. 01, 1735 (B), and Jul. 01, 1735 (C).

### Regression Analysis

Table 2 shows the results of the regression analysis. The proportional regression model (Model I) suggests that in the period A that preceded the 1729–34 crisis no category exhibited significantly different mortality when compared to the reference category I. Although, children conceived in the peak of the measles epidemic (category IV) had been characterized by a relatively high hazard ratio (HR = 1.433; p>.10). The fixed-effect model (Model II) suggests that those in category IV have significantly increased mortality when compared to reference (HR = 3.030; p<.05), if the model considers child’s family membership.

**Table 2:** Results of the Cox regression models, child mortality between Jan. 01, 1729 & May 31, 1729 (A), between Jun 01, 1729 & Jun. 30, 1734 (B 2^nd^ crisis) and between Jul. 01, 1734 & Dec 31, 1740 (C).

Mortality during period	A (Jan. 01, 1725–May 31, 1729)		B (2^nd^ crisis) (Jun. 01, 1729–Jun. 30, 1734)		C (Jul. 01, 1734–Dec. 31, 1740)	
Type of Model	I (standard)	II (FE)	I (standard)	II (FE)	I (standard)	II (FE)
N individuals	5922	1186^a^	5675	1466^a^	5330	1091
N families (N strata)	−	220	-	278	-	218
N deaths	247	247	345	345	250	250
**Exposure status**		***				
**I** conceived & born before the epidemic (REF) [born Jan. 01, 1705–Dec. 31, 1713]	1	1	1	1	1	1
**II** conceived before, born in the epidemic [born Jan. 01, 1714–Aug. 31, 1714]	0.482	0.716	1.112	1.234	0.795	0.6287
**III** conceived in the start, born in the epidemic [born Sep. 01, 1714–Feb. 28, 1715]	0.593	0.596	0.891	0.627	0.805	0.684
**IV** conceived in the peak, born after the epidemic [born Mar. 01, 1715–Jul. 31, 1715]	1.433	3.030*	0.103*	0.137+	0.920	0.768
**V** conceived in the end, born after the epidemic [born Aug. 01, 1715–Nov. 30, 1715]	1.108	0.929	0.504	0.780	1.325	1.260
**VI** conceived & born after the epidemic [born Dec. 01, 1715–Dec. 31, 1724]	0.627	0.801	0.964	0.995	1.102	0.956
**Control variables**						
Mother’s age at death	1.003	0^a^	0.997	0^a^	0.988**	0^a^
Father’s age at death	0.991+	0^a^	0.998	0^a^	0.992+	0^a^
Sex (female)	0.965	1.063	1.032	0.992	1.415**	1.576**
Birth rank	0.962	1.028	0.998	1.083	1.060+	1.053
Mother residential status						
1) place of marriage and death identical (REF)	1	0^a^	1	0^a^	1	0a
2) place of marriage, birth and death identical	0.753	0^a^	0.948	0^a^	1.117	0a
3) place of marriage and death not identical	0.853	0^a^	1.364+	0^a^	1.062	0a
Father’s residential status			**			
1) place of marriage and death identical (REF)	1	0^a^	1	0^a^	1	0a
2) place of marriage, birth and death identical	1.084	0^a^	0.791	0^a^	0.877	0a
3) place of marriage and death not identical	0.963	0^a^	0.544***	0^a^	0.994	0a
Maternal age at birth						
>14–19	0.864	0.796	1.041	0.938	1.566	1.525
>19–25	1.006	1.155	1.084	1.059	0.942	0.901
>25–35 (REF)	1	1	1	1	1	1
>35–40	1.333	1.669	1.022	1.001	1.039	1.487
>40–45	1.725*	2.152	0.980	0.998	0.741	1.064
>45–50	0.644	0.547	0.000	0.000	0.657	1.453
Child’s age at Jan. 01, 1725 (A); Jun. 01, 1729 (B); Jul. 01, 1740 (C)	0.664***	0.692***	1.046	1.094	1.413**	1.432*
Child’s age at Jan. 01, 1725 (A); Jun. 01, 1729 (B); Jul. 01, 1740 (C) SQUARED	1.018***	1.019***	0.999	0.999	0.993**	0.992*

Models of type I (standard) are proportional Cox regression models; Models of type II (FE) are Fixed-Effects Cox regression models that control for unobserved family characteristics with fixed effects for the marriage.

a –Degree of freedom reduced as compared to corresponding model I because of constant or linearly dependent covariates due to stratification.

***p<0.001;

**p<0.01;

*p<0.05;

+p<0.1.

In the period B that is the second crisis years 1729–34, mortality among those who were conceived during the peak of the 1714–15 epidemic (category IV) decreased sharply (HR = 0.103, p<.05 in the multivariate adjusted model; HR = 0.137, p<.10 in the fixed effects model). These differences further support the idea that those who were conceived during the peak of the epidemic have lower mortality in later crisis years. For those who were conceived during the early (category III) or late phase of the epidemic (category V) mortality not significantly different from the reference group.

Results for the period C following the 1729–34 mortality crisis do not suggest any significant differences in mortality by earlier epidemic exposure status.

Results for the control variables are mostly in the expected direction or not statistically significant. For example, depending on the model, mother’s and father’s age at death are either negatively associated with mortality or not statistically significant, and birth rank and mother’s age at birth are positively associated with mortality or not statistically significant.

## Discussion

The concept of functional trans-generational mechanisms which responds to pathogen stress predicts that individuals who were conceived under a high disease load are less vulnerable in time periods with an increased disease load, but face a phenotype-environment-mismatch that might include increased mortality when disease load is low. Our results from the historical population of the St. Lawrence valley are consistent with this prediction. We found that children who were conceived during the peak of the 1714–15 measles epidemic had increased mortality 10 years later in a period of low mortality (1725–29) but exhibit decreased mortality in a later crisis period that occurred in 1729–34. The results suggest that trans-generational effects affected the development of offspring’s phenotype that would be functional under high levels of epidemic stress, but which would not increase or even decrease survival probabilities if the disease load were low.

Our results support the existence of functional trans-generational response mechanisms in humans. However, our study design does not allow investigation of the underlying proximate mechanisms since biometric measurement or molecular-biological information is not available. We can therefore only speculate on the physiological modifications as well as on the transmitting molecular-biological mechanisms. It is possible that the parental information about the predicted high pathogen environment is transmitted via epigenetic pathways to the offspring; e.g. via modified de-novo-methylation of the DNA of the oocytes [27]. This causes an increased resource investment in the immune system of the developing offspring. The augmented investment in the immune system in turn might be at the expense of other developmental processes and could explain why mortality is increased in the low pathogen period (phenotype-environment-mismatch).

In addition to trans-generational effects, there are competing explanations for the mortality patterns found. There are several selective mortality scenarios that might be responsible for our findings. First, it is possible that the cohort that was conceived during the epidemic is more robust due to selection by parental characteristics. Our analysis compared siblings who were born to same parents, ruling out the selection by parental characteristics explanation. Selective mortality due to the measles epidemic of 1714–15 leading to better later survival is also unlikely because of two reasons. First, more robust individuals should exhibit lower mortality not only in crises, but also in non-crisis years; however, the cohort that was conceived during the peak of the epidemic did exhibit lower not higher survival rates in preceding non-crisis years and no significantly different mortality in years that followed the crisis of 1729–34. We also investigated the life expectancy after age 30 (data not shown). We did not find that an individual’s chances of survival later in life were affected by the exposure status during conception. Second, the selection should have taken place intrauterine, which would have resulted in increased miscarriages and decreased numbers in live births [28]–[31]. However, based on the monthly numbers of births between 1710 and 1720, we did not find evidence of decreases in births during the 1714–15 epidemic (see Figure 1).

It is also possible that selective mortality is causing the decreased mortality during the second mortality crisis in 1729–34 among the children who were conceived in the peak of the 1714–15 epidemic. High intrauterine disease load may increase post-birth mortality [28], and we observed that those conceived in the peak to the 1714–15 epidemic had increased mortality before the second mortality crisis in 1729–34 crisis period. However, children among category III had also been exposed to high levels of pathogen intrauterine, but they do not exhibited increased mortality in the period prior the second crises. Further, the prevalence of measles during childhood prior to the development of a vaccine in the 20^th^ century makes it likely that the majority of adult individuals were at some point exposed. If so, many mothers not only benefited from lifelong immunization, but also that their immune system was able to shield the fetus against intrauterine measles exposure [32]. Although Mazan[17] reports that the epidemic of 1714–15 was the first confirmed measles epidemic of New France, many parents at the beginning of the 18^th^ century had been born in the Old World and/or could had have contact with the virus not in the context of an epidemic. Moreover, among the children who were conceived in the peak of the 1714–15 epidemic mortality in the 2^nd^ crisis period in 1729–34 is decreased so much that even if we moved all the excess deaths observed in the period preceding the second crisis, their mortality in years 1729–1934 would still be lower than expected.

A second alternative explanation is an “acquired vulnerability scenario” in which the results are driven by increased mortality in the reference category, not decreased mortality among those who were conceived during the peak of the 1714–15 epidemic. This hypothesis is supported by the observation that a measles infection may be accompanied by severe complications (e.g., croup, bronchitis, bronchiolitis, pneumonia, conjunctivitis, myocarditis, hepatitis, encephalitis), which may in turn have long-term negative consequences [32]. This might be the reason for the finding that mortality among the children who survived the measles was often higher later in life [28], [33], [34]. While we should not ignore these findings, we do not believe that the reference category in our study suffers from an acquired vulnerability. If this was the case, however, each cohort who had contact *ex utero* with the measles epidemic of 1714–15 should have been affected. However, the opposite was observed. The children who were born during the measles epidemic exhibited lower mortality rates in the period before the second crisis when compared to those who were born before the 1714–15 epidemic. Furthermore, it may be expected that who were born during the measles epidemic would have faced measles-related complications and their long-term consequences, because they were younger when exposed to the measles than those in the reference category. Therefore, they should have been more, not less, vulnerable. In addition, those who were conceived during the peak of the epidemic had a lower mortality relative to the reference category only during the second crisis; if the results were driven by the reference category being more vulnerable, we should have observed this difference also in non-crisis years. However, this was not observed. Finally, Mazan [33] reported that survivors of the measles epidemic of 1714–15 suffered from delayed mortality up to the age of three but not later. We therefore excluded the birth years 1711–13 from the references category. The results of the models changed only marginally (data not shown).

A third alternative to our trans-generational effect interpretation is a “costless immunization scenario.” A measles infection results in lifelong immunization, but it may also be associated with long-term negative health consequences. We also know that breastfed children born to immunized mothers are protected by maternal antibodies in the breast milk [32]. Based on these considerations, two different immunization scenarios are possible. First, children who were exposed to the measles epidemic after weaning faced the disease without protection, and ran the risk of suffering long-term negative consequences. In contrast, children who were protected intrauterine and/or during lactation by maternal antibodies may have been immunized and therefore did not come down with the measles. In other words, the immunization would have been “costless” for these children. An individual’s survival in a later epidemic might have also depended on how the child became immunized. The problem with a costless immunization scenario is that it does not explain why measles-immunized individuals were better off in the later smallpox epidemic which consisted a large part of the 1729–34 crisis.

The scenarios discussed above are all based on the assumption that the exposure of children to the measles epidemic of 1714–15 was directly linked to later mortality differentials. However, there is an alternative explanation scenario that does not necessarily assume that the exposure to the measles itself affected later survival. In this scenario, children who were conceived during the peak of the epidemic (note that those have been born after the measles epidemic) benefited from a long inter-birth interval that was extended by the measles death of their nearest elder sibling. This might have increased the resources available for the surviving child, which would have increased later survival [35]–[37]. It is expected that the lower the interbirth interval, the higher the effect. As we noted above, the population of Québec was naturally fertile and had rather low infant and child mortality rates, a combination which resulted in exponential growth. The interbirth intervals were also low (on average, 1.88±1.49 years between the first and second births, 1.90±1.09 years between the second and third births, and 1.91±0.97 years between the third and fourth births). In order to test this hypothesis, we included a dichotomous variable in the models containing the information about whether the nearest sibling died during the measles epidemic. The results changed only marginally (data not shown).

In summary, alternative explanations to the observed mortality patterns are problematic. We conclude that functional trans-generational response mechanisms may be responsible for these findings. This has a number of implications for public health. First, a rethinking of our perspective on populations that are under permanently high levels of epidemic stress is necessary. The functionality and efficiency of the individual immune system–and, consequently, of a person’s health and mortality–would not depend only on individually experienced factors, like nutritional conditions, but also on the parental environment. This revised perspective would also include phenotype-environment-mismatch scenarios, in which the future environment differs from that prevailing during conception and results in decreased fitness. Under this mismatch scenario rapid environmental improvement may not always be beneficial if the early life or intrauterine experience differs markedly from the later environment. Second, it may be expected that the initiation of the trans-generational pathogen response mechanism will occasion costs, even under an optimal phenotype-environment-match. Resources that are increasingly invested in the immune system might not be invested in other domains, such as growth. Conversely, when intrauterine disease load is reduced, this may lead to reduced trans-generational pathogen response and could allow more to be invested in growth. This could potentially explain part of the secular increases in body size and earlier puberty.

While our results suggest that functional trans-generational response mechanisms operate among humans and influences their mortality, an important limitation in our analysis was the sample size. While those who were conceived during the peak of the epidemic had remarkably low mortality in later crisis years when compared to other cohorts (mortality hazard ratio less than.2 across all models), we observed only 140 individuals from this cohort during the later crisis and only one of these died during the period. Further analysis with larger cohorts are needed to test the robustness of our finding.
